# Decoding ALS from the tail end of RNA

**DOI:** 10.1016/j.xgen.2025.101102

**Published:** 2025-12-10

**Authors:** Yusuke Fujioka, Shinsuke Ishigaki

**Affiliations:** 1 Molecular Neuroscience Research Center, Shiga University of Medical Science, Otsu, Shiga 520-2192, Japan

## Abstract

In this issue of *Cell Genomics*, McKeever et al.^1^ generate a single-nucleus transcriptomic atlas of ALS/FTLD brain and reveal widespread alternative polyadenylation changes. Their findings highlight 3′ end RNA processing as a central integrator of stress responses, cell-type specificity, and disease susceptibility, offering new mechanistic insight and potential therapeutic directions.

## Teaser

In this issue of *Cell Genomics*, McKeever et al. generate a single-nucleus transcriptomic atlas of ALS/FTLD brain and reveal widespread alternative polyadenylation changes. Their findings highlight 3′ end RNA processing as a central integrator of stress responses, cell-type specificity, and disease susceptibility, offering new mechanistic insight and potential therapeutic directions.

## Main text

Amyotrophic lateral sclerosis (ALS) and frontotemporal lobar degeneration (FTLD) are devastating neurodegenerative disorders that share overlapping clinical, genetic, and molecular features. A growing body of evidence points to a common pathological denominator: the breakdown of RNA metabolism caused by dysfunctional RNA-binding proteins (RBPs) such as TDP-43, FUS, and others. These proteins regulate multiple layers of RNA processing, including splicing, transport, and translation, and their mislocalization triggers a cascade of downstream transcriptional and post-transcriptional disturbances.

**Figure 1:**
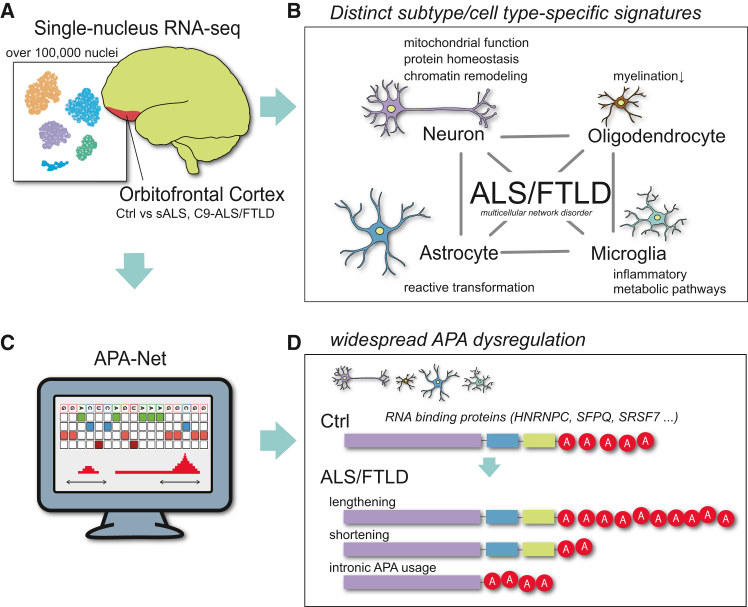
Cell-type-specific transcriptional and 3′ end RNA remodeling in ALS/FTLD (A) snRNA-seq of over 100,000 nuclei from ALS/FTLD orbitofrontal cortex reveals broad transcriptional and post-transcriptional changes. (B) ALS/FTLD emerges as a multicellular network disorder: neurons show disrupted mitochondrial function, proteostasis, and chromatin remodeling, while glial cells exhibit distinct responses—microglial activation, astrocytic reactivity, and oligodendrocyte demyelination. (C and D) Widespread alternative polyadenylation (APA) shifts occur in a cell-type-dependent manner, generating transcript isoforms with variable 3′ UTRs. Deep-learning analysis (APA-Net) identifies RBPs such as HNRNPC, SFPQ, and SRSF7 as candidate regulators. These findings highlight APA as a key regulatory layer linking RBP dysfunction to ALS/FTLD pathogenesis.

Among them, TDP-43 mislocalization is a pathological hallmark, yet they differ in their affected regions and clinical phenotypes.^1^^,^^2^ In this issue of *Cell Genomics*, McKeever et al.^1^ extend our molecular view of ALS/FTLD beyond the motor cortex by constructing a single-nucleus RNA sequencing (snRNA-seq) atlas of the orbitofrontal cortex, a region crucial for cognition and behavior. Profiling over 100,000 nuclei from C9orf72-linked and sporadic ALS/FTLD brains, the authors reveal how cell-type-specific transcriptional and post-transcriptional mechanisms converge to shape disease heterogeneity (Figure 1A).

Neurons emerged as the most vulnerable population, exhibiting broad changes in mitochondrial metabolism, proteostasis, and chromatin remodeling. Upregulation of STMN2 and NEFL, key mediators of axonal maintenance, was a consistent adaptive signature to TDP-43 dysfunction. In parallel, glial cells followed distinct activation trajectories: microglia upregulated inflammatory and metabolic pathways, astrocytes underwent reactive transformation, and oligodendrocytes suppressed myelination genes. These data emphasize that ALS/FTLD is not a neuron-centric disease but a multicellular network disorder in which diverse cell types engage in maladaptive communication (Figure 1B).

Crucially, McKeever et al. identify alternative polyadenylation (APA) as a pervasive and cell-type-dependent regulatory layer.^1^ APA generates transcript isoforms with variable 3′ UTR lengths, influencing RNA stability, localization, and protein output. Thousands of APA shifts—often toward distal sites and longer 3′ UTRs—were detected independently of changes in gene expression. This finding elevates 3′ end remodeling from a passive byproduct to an autonomous regulatory mechanism. Notably, dysregulated APA has been implicated in other neurodegenerative disorders, suggesting a convergent theme across diseases^3^^,^^4^ (Figure 1C).

By integrating APA dynamics with RBP expression, the authors highlight candidate regulators, including HNRNPC, SFPQ, and SRSF7, proteins already linked to ALS/FTLD through TDP-43-associated RNA dysregulation.^5^^,^^6^^,^^7^ This alignment of computational prediction and pathological evidence underscores how altered RBP activity, through aggregation or sequestration, can rewire transcript-end selection. Mapping these RBP-APA networks in human brain tissue provides a mechanistic bridge between molecular pathology and cell-type-specific vulnerability (Figure 1D).

The study also prompts broader reflection on transcriptional kinetics and chromatin architecture, both of which may shape APA site choice. Future integration of APA signatures with ATAC-seq or single-cell chromatin accessibility maps could reveal whether RNA-tail remodeling acts as an early trigger or a downstream compensatory response to cellular stress.^3^^,^^4^ Such multi-omic frameworks may illuminate how RNA-processing decisions are coupled to nuclear architecture and neuronal activity.

Importantly, this resource opens opportunities for translational applications. The identification of cell-specific APA patterns and their regulatory RBPs offers potential biomarkers for disease staging and progression. Moreover, the possibility of modulating APA or RBP activity raises the prospect of restoring healthy isoform balance, an emerging therapeutic strategy already gaining traction in antisense and small-molecule approaches.^5^ The data therefore provide not only a molecular atlas but also a conceptual roadmap for targeting post-transcriptional regulation in ALS/FTLD.

While this study provides an unprecedented cell-type-resolved view of alternative polyadenylation, several questions remain open. The mechanisms linking APA shifts to neuronal dysfunction are largely correlative, and experimental validation in model systems will be essential to establish causality. Moreover, how these APA changes evolve during disease progression or in response to therapy remains to be determined.

By illuminating how the 3′ ends of RNA dynamically respond to neurodegenerative stress, McKeever et al. invite a paradigm shift: the RNA tail is not merely a terminus but a platform for regulatory decision-making.^1^ Their integrative analysis positions APA as a frontier for understanding, and eventually correcting, the complex RNA landscape of ALS/FTLD and related disorders. Looking ahead, this study paves the way for future mechanistic and translational research that will deepen our understanding of RNA homeostasis and open new avenues for therapy.

## Declaration of interests

The authors declare no competing interests.

## Declaration of generative AI and AI-assisted technologies in the writing process

During the preparation of this manuscript, the authors used a combination of Gemini 2.5 Pro (Google) and ChatGPT-5 (OpenAI) for grammatical error correction and stylistic changes. The authors thoroughly reviewed and edited the content following the use of these tools and take full responsibility for the final content of the publication.
